# Hotspot analysis for organic laying hen husbandry—identification of sustainability problems as potential risk points to lose consumers’ trust

**DOI:** 10.1007/s13165-023-00426-5

**Published:** 2023-03-28

**Authors:** Elisa Bayer, Marie von Meyer-Höfer, Sarah Kühl

**Affiliations:** 1 Marketing for Food and Agricultural Products, Georg-August Universität Göttingen, Platz der Göttinger Sieben 5, 37073 Göttingen, Germany; 2 Thünen Institute for Market Analysis, Bundesallee 63, 38116 Braunschweig, Germany

**Keywords:** Organic livestock farming, Organic laying hen husbandry, Animal welfare, Environmental impact, Consumer expectations

## Abstract

Over the last decade, there has been growing societal concern about the welfare of farmed animals. Although organic agriculture provides higher living standards, there are still critical points which can damage consumers’ trust in organic livestock farming. That is a risk, as especially organic farming relies on consumer trust. A hotspot analysis was conducted to identify critical points within the organic laying hen husbandry in Germany. This methodology aims to examine the sustainability of a product along its whole life cycle. Based on literature reviews, the life phases *breeding*, *keeping*, *feeding*, *animal health*, *transport*, and *slaughter* were assessed with ecological, social, and animal welfare criteria. Finally, the results were triangulated with various experts, and the critical points were classified in terms of their potential to diverge from consumers’ expectations. Our results show a high dependency of the organic sector on the conventional breeding process and its specialized breeds. This fact involves critical points which contradict the ideology of organic farming. The loopholes in the organic EU regulations in transport and slaughter were identified as additional threats to consumer trust in the organic system. The overall not better animal health compared to the conventional poultry system and the high numbers of poultry kept on some organic farms are also possible causes of disappointment in consumers’ vision of organic livestock farming. Therefore, we recommend an adjustment of some organic EU regulations regarding these points. Further, a linkage of the organic certification of a slaughterhouse to higher animal welfare standards during slaughter should be considered.

## Introduction

Over the last decade, there has been a change in the society regarding how animals are perceived, and thus the expectations of how animals should be kept and treated have increased (Otterstedt 2012; BMEL 2014; WBA 2015; Busch et al. 2015). Hölker et al. (2019) show that the long-existing anthropocentrism, where humans are allowed to use animals as they wish, has been replaced by the contractarian approach with the understanding of a “fair deal.” In other words, animals can be used as long as humans ensure them a good life. The effects of increased animal welfare concerns in society are reflected in the refusal of long-established common practices, such as the culling of day-old males from laying hens, the castration of piglets without anesthetization, or non-curative interventions such as beak trimming, which are current subjects of public discussions (Zander et al. 2013; Busse et al. 2019).

Animal welfare is defined as the physical health of an animal as well as its well-being, resulting from the ability to successfully interact with its environment, leading to positive emotions (Knierim 2001). Organic husbandry conditions are clearly beneficial to the latter aspect of animal welfare (March et al. 2019; Iannetti et al. 2020), whereas the physical health state of organically farmed animals in some areas such as keel bone deformities (Wilkins et al. 2011; Riber and Hinrichsen 2016), parasite infection (Sharma et al. 2019; El Jeni et al. 2021), and mortality (Weeks et al. 2016; March et al. 2019) is often not better than that on conventional farms. Furthermore, also in organic egg production, the male chicks from laying hens are culled right after hatching (in Germany this practice was banned in 2022) (BMEL 2022). This background shows the need to discuss animal welfare concerns, even in organic livestock farming.

Especially for consumers of organic animal products, animal welfare is one of the main reasons to buy organic (Zander and Hamm 2009; von Meyer-Höfer et al. 2015; Lee and Yun 2015; Ökobarometer 2017). For example, Heid and Hamm (2013) found regarding the specific topic of piglet castrations (which was allowed till 2012 in organic farming without pain relief) that organic consumers prefer alternatives with higher animal welfare and show a higher willingness to pay for the more animal-friendly alternatives—showing that animal welfare and treatment is an important issue for organic consumers. Additionally, the review from Schleenbecker and Hamm (2013) on consumers’ perception of organic products concludes that taste, nutritional quality, and health are the most important aspects directly related to an organic product. Further environmental concerns, the absence of pesticides, and animal welfare are important in a broader perspective. However, most characteristics of organic products, such as higher animal husbandry standards or the lack of pesticides, cannot be examined by the consumers themselves.

These are so-called credence attributes (Dahlhausen et al. 2018) and therefore trust is inherently important for purchasing organically produced products (Pivato et al. 2008; Spiller and Codts 2010; Nuttavuthisit and Thøgersen 2017). Trust is characterized by a state between knowing and not knowing and serves to overcome knowledge or information deficiencies (Zagata and Lostak 2012; Tonkin et al. 2015). Studies show that most consumers have little knowledge about the standards of organic farming and animal husbandry in general (Pivato et al. 2008; Janssen and Hamm 2011; Zagata und Lostak 2012; Di Pasquale et al. 2014). Organic labels can serve as a source of trust (Hamzaoui-Essoussi et al. 2017) but are also linked to high expectations as shown above. Failure to meet these consumer expectations can cause lasting damage to the organic industry, as, according to Nocella et al. (2010), trust is based on consumers’ expectations. If these expectations are not met in reality, this can lead to a feeling of disappointment and according to Möllering (2008) also to a loss of trust. Moreover, Wu et al. (2021) found that higher trust levels lead to higher confidence that products comply with expected standards. Distrust, in contrast, is associated with lower expectations and decreases the buying intention and willingness to pay for the labeled product (Nuttavuthisit and Thøgersen 2017; Canova et al. 2020).

Thus, the identification of critical points in terms of sustainability weaknesses (referring to ecological, social, and animal welfare aspects) is of high importance for the organic sector in order to meet its own and society’s demands for more sustainable animal husbandry and to justify the trust placed in the organic industry and therewith keep the consumers’ willingness to buy organic products in the long term. The hotspot analysis (HSA) conducted in this study investigates and evaluates the sustainability of organic laying hen husbandry along the entire production process by means of a literature review (Wallbaum and Kummer 2006; Bienge et al. 2009). Subsequently, interviews with different experts supplement our results of the literature review. Many problems and challenges in organic animal husbandry are certainly known. However, the aim of the HSA is to present these problems in an overview from breeding to slaughter. Furthermore, a first assessment of the identified hotspots will be given with regard to a possible trust risk for consumers. Detailed research on consumer expectations of organic livestock production is rare and even non-existent for some aspects. Therefore, it is not possible to classify the sustainability hotspots in this analysis with certainty as points of consumer trust loss. For this reason, we have classified them as “potential risk points to lose consumers trust” because the consumer side is not well documented and reported. For this reason, the classification is based on literature regarding consumers’ general attitude toward organic farming and expert’s assessment.

Organic laying hen husbandry was chosen as the scope for this HSA, as this is where we expected the most challenges and problems due to the high degree of specialization in breeding and husbandry (Hammershøj et al. 2021) and feeding challenges as monogastric animals (van Krimpen et al. 2016). Further eggs are one of the most commonly bought organically produced foods in Germany (Ökobarometer 2019), with their market share constituting 11.4% of the total German egg production (BÖLW 2020). Organic egg production is thereby twice as high as the total market share for organic products, which is 5.6% (Statista 2020a). The aim of this HSA is to provide an overview of the weaknesses and loopholes along the entire production cycle of organic laying hen husbandry kept according to organic EU regulations (EU-Öko-VO 2018). The identified hot spots can be seen as possible risk points, which, however, by no means exist everywhere in organic laying hen husbandry, due to high variability of farming and management practices (WBAE 2020). The hotspots can be seen as starting points to improve sustainability in some areas and to integrate consumers’ expectations in the prospective development of these domains, as well as to build a suitable method of communication to strengthen costumers’ trust in organic livestock farming.

## Methods

A HSA was conducted to identify critical points in terms of sustainability according to different criteria referring to ecological, social, and animal welfare aspects such as biodiversity, working conditions, or animal welfare (see Table 2. This methodology aims to investigate the sustainability of a product along its entire production cycle by means of a literature review (Wallbaum and Kummer 2006; Bienge et al. 2009; Liedtke et al. 2010).

**Fig. 1:**
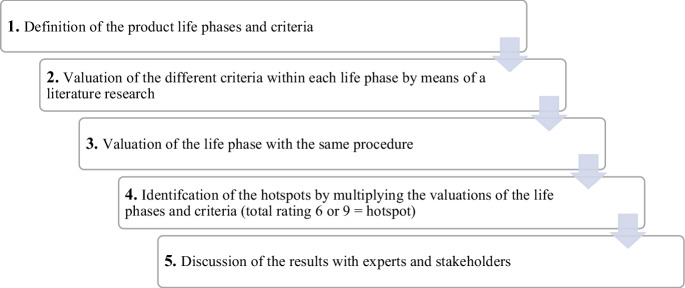
General procedure of the HSA according to Wallbaum and Kummer 2006; Liedtke et al. 2010; Schmitt and Hamer 2018

The hotspots are areas with sustainability weaknesses, for example, poor biodiversity. The HSA systematically visualizes these hotspots but gives no information about their reduction potential (Wallbaum and Kummer 2006). The general HSA procedure contains five steps, which are shown in Fig. 1.

The assessment by literature review ranges from a score 1 to 3. Score 1 represents a low relevance, and no problems were found. An assessment of 2 defines areas with some problems in terms of sustainability, but solutions and advantages also exist. An assessment of 3 represents an area with high relevance and sustainability problems. The assessment of the life phases and the criteria get multiplied to identify the hotspots. Thus, the total rating is the multiplication of the life phase and criteria ratings. For example, if a life phase receives a rating of 3 and a criterion is allocated a rating of 2, the multiplication of these ratings results in a total of 6 for this criterion in this life phase. Criteria that reach total values of 6 or 9 are classified as hotspots (Liedtke et al. 2010).

### Scope of the HSA for organic laying hen farming

In the following, the scope of the HSA will be defined. This HSA refers to organic laying hen husbandry in Germany. Overall organic farming represents a holistic approach to farming by trying to combine food production with environmentally friendly procedures, high biodiversity, protection of resources, and high animal welfare (EU-Öko-VO 2018). The EU regulations serve as a benchmark for organic farming because they define the minimum standards of organic agriculture. Nevertheless, in Germany, about 50% of organic farms are additionally certified by an organic association like Bioland, Naturland, and Demeter, which have even higher standards (Statista 2020b). Since 1.1.2022, a new EU regulation came into place. It holds stricter and new regulations in some areas. The main changes for laying hen husbandry concerning the purchase of conventional protein feed (5% of the total ration) for poultry, which is now only permitted in rearing. Further, the acquisition of conventionally raised animals is more restricted, and now so far missing regulations concerning husbandry conditions of young hens, male layers, and parental animals were introduced. These EU regulations merely present the minimum of compliance in organic poultry farming. There is a high variability in terms of husbandry systems and management. The purpose of this HSA is to provide a general overview of the problems that can exist in organic laying hen husbandry, because the differences in keeping and management cannot be considered. Further, the focus lies only on areas that are connected directly to the life phases or criteria. Up- and downstream processes are not considered.

### Definition of life phases and criteria

The life phases of organic laying hen farming were defined as shown in Table 1. Not every life phase which is described here can be seen as such. For example, *animal health* or *feeding* are not biological phases in an animal’s life, but they are considered important areas in terms of external effects. Therefore, they are defined as a “life phase” for this investigation.

**Table 1:** Defined “life phases” of organic laying hen farming with description

Life phase	Description
Breeding	Breeding process, selection traits
Keeping	Organic keeping regulations, housing systems
Feeding	Feed production, purchase regulations, feed ration composition
Animal health	State of health, regulations for medical treatments, non-curative interventions
Transport	Transport organization, loading/unloading, handling
Slaughter	Arrival at the slaughterhouse, handling, stay in the waiting area, stunning, killing

Source: own description

In terms of the investigation criteria, the ecological and social criteria according to Bienge et al. (2009) were used, albeit in a slightly modified version, for organic laying hen husbandry as shown in Table 2. The adjustments concern the criteria *waste*, *raw material*, and *animal welfare*. Due to the investigation’s subject of organic laying hen farming, the criterion *waste* also included *by-products* (*side products of a process with no specific use*), and the criterion *raw material* was changed to *breeds/genetic lines used* including the *breeding process*. As animal welfare is an especially important criterion for organic poultry farming, it received its own category. Special attention was paid to it because animal welfare presents a variable with high relevance to assess agricultural sustainability (Haller et al. 2020). Table 3.

**Table 2:** Criteria according to Bienge et al. (2009) with adjustments for organic laying hen husbandry differentiated by three categories: ecological criteria, social criteria, and animal welfare

Ecological criteria	Social criteria	Animal welfare
**Breeds/genetic lines used** Used breeds/genetic lines, breeding process	**Working condition** Working hours, contracts, accommodation, occupational safety	**Animal welfare** Animal well-being
**Energy** Electricity, feeds energy content	**Social stability** Social security, insurance	**Animal protection** Compliance with existing regulations
**Greenhouse gas emissions** Carbon dioxide, methane, nitrous oxide	**Education and training** Qualified workers, know-how, education	
**Water consumption** Water demand	**Occupational health** Hygiene, contact with contaminants and pathogens	
**Land use** Land demand	**Human rights** Exploitation, discrimination	
**Biodiversity** Diversity of species, genetic diversity	**Income** Sufficient income, minimum wages	
**Water, soil, and air emissions** Nitrate, fertilizer, chemicals, smell, dust, methane	**Consumers’ health** Product safety, contamination with pathogens, transparency	
**Waste, by-products** Unusable products, animals	**Product quality** Taste, smell, ingredients, consistency, appearance, shelf life	

Modified to organic laying hen husbandry after Bienge et al. (2009)

### Literature research and expert interviews

Literature research was carried out to evaluate the life phases and the criteria. The search engines Google and Google scholar were used as well as the databases Organic Eprints, ScienceDirect, and Web of Sciences. Furthermore, literature that originated from references of already detected literature was included. First, each life phase is described, followed by systematically identified sustainability problems based on the ecological and social criteria and the criterion of animal welfare.

The search keywords used were the criterion in combination with the respective life phase, such as “water consumption AND slaughter.” Occasionally the search terms “organic laying hens” were added. Also, some more specific terms concerning the life phases were used, e.g., plumage condition or parasite infestation in the life phase *animal health*. The web-based search was focused on scientific papers, conference papers, final reports of relevant projects, leaflets, and guidelines, as well as dissertations. Newspaper reports were also referred to for current issues such as poor working conditions in the slaughter industry. Especially in the life phases of *transport* and *slaughter*, relevant reports from animal welfare organizations such as Animals’ Angels or the Albert Schweitzer Foundation were also included. Moreover, relevant literature had to meet at least the following criteria: They had to be published in or after 2000, the results had to refer to Europe and in particular to Germany, and they had to be written in German or English.

In the last step, the identified hotspots were discussed with nine different experts and stakeholders from the organic sector. The experts were classified into three main groups (3 experts per group): scientists (professors), practitioners (farmers, consultants), and opinion leaders (journalists, authors, politicians). All experts worked for several years in their field. Table 4 in the Appendix gives a more detailed overview of the expert’s field of expertise. The interviews took place via Zoom. At first, the results of the HSA were presented to the experts, and the following leading questions were asked:

1. Would you change any of the valuations? Did we miss an important point?
2. Which hotspots would you classify as the ones most at risk of losing consumers’ trust?

The evaluation of the expert interviews was performed by a qualitative content analysis. A short summery of the main statements is given at the end of the results chapter.

## Results

### Evaluation of the life phases and criteria

First, each life phase will be described, and their rating will be discussed briefly. The identified hotspots will be described according to the life phases they belong to. Hot spots with high ratings (rating life phase: 3 and rating criterion 3 = total rating: 9) get a more detailed description than hot spots with lower ratings (6). The ratings of the hot spots are displayed after the respective criteria.

#### Breeding- rating 3 (high relevance)

Poultry breeding is a highly specialized process. Modern meat and egg production are characterized by the use of high-performance hybrid breeds (Cheng 2010; Athrey 2020; Scanes et al. 2020). Thereby, specialized genetics are used both in egg and in meat production (Scanes et al. 2020). Despite the intention of EU regulations for organic agriculture to use robust and adapted breeds (EU-Öko-VO 2018), organic poultry farming also relies heavily on these specialized high-performance genetics (Baldinger n.d.; Gura 2010; Hörning et al. 2011; Ökolandbau 2018a). This applies more to egg than to meat production. In broilers, the use of slow-growing hybrid lines (60% growth potential of conventional fatting chicks) is widespread (Schmidt and Bellof 2008; Schaack et al. 2018). The use of specialized hybrid breeds contradicts the holistic concept of organic farming and raises some ethical issues. Thus, although some programs and projects deal with special organic breeds (Ökologische Tierzucht gGmbH 2016; Universität Bonn Landwirtschaftliche Fakultät 2020), up until now, these breeds constitute a niche within organic poultry farming (Preisinger 2016; Deutscher Bundestag 2020). For example, out of 5.3 m. organic laying hens, 110,000 are form dual purpose breeds originating from the Ökologische Tierzucht GmbH (Ökolandbau 2021). The life phase *breeding* received a rating of high relevance (3) because breeding impacts several areas and affects the animals’ life to a high degree.

##### Hot spots in the life phase breeding (rating)

Five hotspots were found for the life phase *breeding*, which are described below:

- ***Breeds/genetic lines (6):*** Dependency and monopolization in animal breeding (Idel n.d.; Feindt et al. n.d.; Flock et al. 2008; Gura 2015; WBA 2015). In recent years, organic farming is establishing its own breeding programs (Ökologische Tierzucht gGmbH 2016).
- ***Land use (6):*** Slow-growing lines and dual-purpose breeds are less resource-efficient (Schmidt and Bellof 2008; Lohmann Tierzucht GmbH 2013; Schütz et al. 2018); therefore, a large-scale use of these animals is only possible in conjunction with a reduction in the consumption of animal products (Muller et al. 2017). In general, organic farming follows a holistic concept, for example linking animal husbandry to field area, which reduces the environmental impact (Kratz et al. 2004; Castellini et al. 2006; Umweltbundesamt 2020).
- ***Biodiversity (9):*** In the life phase *breeding*, biodiversity refers to the genetic diversity of different breeds and lines as well as the genetic variability within the breeds (Schulte-Coerne et al. 2014). With the emergence of modern and high-performance breeds, most of the old and inefficient breeds have been replaced in agricultural husbandries (Groeneveld et al. 2010; FAO 2015). The breeding process of pure lines, which the high-performance hybrids are based on, causes a further reduction in the genetic variability within the lines (Muir et al. 2008; Gura 2010; ProSpecieRara 2018). According to Muir et al. (2008), there is a reduction in genetic diversity of 50% compared to the original breed. The loss of breeds and genetic variability within the breeds jeopardizes the pool of genetic diversity for future breeding programs (Toro et al. 2009; Groeneveld et al. 2010; Schulte-Coerne et al. 2014). Also, old breeds exhibit valuable traits such as robustness or low demands (Hoffmann 2013; Sosnówka-Czajka et al. 2017) and are predestined for natural husbandry conditions, as organic farming promotes (Sosnówka-Czajka et al. 2017; Ökolandbau 2020a). Therefore, endangered breeds are most likely found on organic farms (Neumann and Rahmann 2001; zu Löwenstein 2019; GEH 2019). However, due to their low performance level, these as well as dual-purpose breeds are not widespread even in organic farming (Preisinger 2016; Deutscher Bundestag 2020; Ökolandbau 2021). Thus, biodiversity also presents a problem in organic laying hen husbandry.
- ***By-products (6):*** The use of conventional high-performance laying hybrids in organic farming leads to a by-product, male chicks, which are commonly killed immediately after hatching (Urselmans and Damme 2014; BMEL 2022). Especially organic farming is looking for solutions to this issue, and different projects are intended: Raising male chicks from layers through subsidies from egg production, usage of dual-purpose breeds, extension of the laying period (Hörning and Häde 2015). Another approach is to determinate the chicks’ sex prior to hatching. Starting at the end of 2021, the killing of day-old male chicks from layers is forbidden by law in Germany (BMEL 2022).
- ***Animal welfare (9):*** An ethical animal welfare issue in laying hens is the killing of male layers as described above. Further, the alignment of the breeding objective which is strongly focused on performance traits leads to breeding-related health disorders for laying hens (Hörning 2017; Jung et al. 2019). There are a series of animal welfare problems that are evoked by breeding. In laying hens, osteoporosis, inflammation of the fallopian tubes, peritoneum, and foot pads, as well as the development of the abnormal behaviors’ feather pecking and cannibalism, are common problems. These diseases are partly caused by genetic predisposition or are favored by the high-performance level of these breeds (Hörning 2017; OMIA 2021). For example, for feather pecking (0.10–0.14) and aggressive pecking (0.27–0.35), low to moderate heritability is reported (Bennewitz et al. 2014; Lutz 2016; Iffland 2021). Stratmann et al. (2016) concluded that selection of specific bone traits associated with bone strength has potential to reduce keel bone damage. Bishop et al. (2000) found for bone strength a heritability of 0.40. Also, Heerkens et al. (2016) confirm a genetic predisposition of keel bone and foot pad disorders in laying hens. Further, high-performance breeds are more susceptible to some infections than animals with a lower specialization on performance (Stehr et al. 2019). However traditional purebred chickens are not automatically more compatible and thus no less susceptible to behaviors such as feather pecking and cannibalism (Ökolandbau 2018b; Hillemacher and Tiemann 2018). Currently, there is a lack of studies that examine how far alternative lines or breeds such as pure breeds or crossbreeds suffer for example from health impairments such as keel bone deformities. The criterion “animal welfare” received a high rating of 3 in the life phase *breeding* because the animals’ well-being is significantly influenced by breeding.

#### Keeping—rating 1 (low relevance)

In total, the life phase *keeping* received a low rating of 1 because the EU regulations for organic animals provide high living conditions compared to the legal requirements, which therefore makes it a comparatively animal-friendly keeping system (TierSchNutztV 2006; EU-Öko-VO 2008; March et al. 2019). Further, the given relation between field area and animal stocking prevents nutrient surplus (Kratz et al. 2004; Umweltbundesamt 2020). Nevertheless, this life phase also has some critical points. One is the number of animals kept on the same farm, which is assessed as too high for consumers and does not correspond with their expectations (Vaarst and Hovi 2004; Chang and Zepeda 2005; Kayser et al. 2012). On average, German farms with organic laying hens keep 10,700 hens, which is, however, half the average number of animals on conventional farms (Statistisches Bundesamt 2018; BLE 2019). Further, the high living standards with access to a free-range go along with higher demands on management. Especially the high nitrogen discharge in the free-range system represents a serious environmental problem. Chickens prefer to stay in sheltered places and especially in the area near the laying hen house, which greatly increases the accumulation of excrement in these areas (Deerberg and Heß 2017; Lüssing-Griese and Gaio 2019). This leads to very concentrated nitrogen inputs of 1500 kg (Deerberg and Heß 2017)–2000 kg n/per ha (Elbe et al. 2005). The high nutrient input through the excrements poses the risk of nutrient leaching and eutrophication of ground and surface waters (Kratz et al. 2005; Elbe et al. 2005; Deerberg and Heß 2017; Lüssing-Griese and Gaio 2019). Therefore, the criterion “water, soil, and air emissions” received a high rating of 3 in the life phase *keeping*. Due to the low rating of the life phase itself, it did not reach the high rating needed to be considered a hotspot.

##### Hot spots in the life phase keeping

No hotspots were found in the life phase *keeping*. Due to the low rating of the life phase itself, the aspect of nitrogen discharge in free-range systems did not lead to the designation as a hotspot. However, it must be considered an environmental problem.

#### Feeding—rating 2 (medium relevance)

Organic feed production uses the concept of a nutrient cycle, avoids pesticides, and follows a field-based animal production, which has positive environmental effects, e.g., benefits to biodiversity (Stein-Bachinger et al. 2019) and less water pollution (FiBL 2012; Kusche et al. 2019). Compared to conventional agriculture, organic agriculture shows higher nitrogen and energy efficiency as well as lower nitrogen balances and nitrogen loss potentials per area (Chmelikova and Hülsbergen 2019).

For poultry feeding, the EU regulations for organic animal farming stipulate that at least 30% of the feed used must come from the same farm where the animals are kept. If this is not possible, the feed should be at least from the same region. This strict regulation contributes less to rainforest deforestation and with this to the displacement of indigenous peoples (Gfbv n.d.; BUND 2019). Due to the rather skeptical attitude of the European population toward green genetic engineering, the clear prohibition of genetic modified crops in organic agriculture can be seen as a benefit to some consumers (Colson and Rousu 2013; Verbraucherzentrale 2018). In terms of sustainability, the higher demand for feed, as well as the lower crop yields in organic agriculture, is a weak point. Organic agriculture has on average 19–25% lower yields than conventional agriculture (De Ponti et al. 2012; Ponisio et al. 2015; Seufert and Ramankutty 2017). According to de Ponti et al. (2012), wheat and barley showed a lower yield gab (< 20%) than soybeans and corn (> 20%). In 2020, the yield gap for organic grain in Germany was on average 52.6% of conventional yield (Ökolandbau 2020b). The yield gap underlies variations that differ between crops and regions (Seufert et al. 2012; De Ponti et al. 2012; Ökolandbau 2020b). Further, organic laying hens have a slightly lower laying performance (2.63–7.5%) compared to conventional farming in Germany (LfL 2012; Destatis 2022). Further, the composition of the feed ration is somehow more difficult; for example, due to the prohibition to add synthetic amino acids (LfL n.d.), these aspects lead to a somewhat higher demand on resources in organic poultry farming; this is especially true for organic broiler as they have a longer fattening period (LfL n.d.; Bellof and Schmidt 2006). In total, organic feed production has a positive impact on several ecological and social criteria but also brings challenges in feeding young monogastric animals according to their needs, and more feed is needed due to lower performance. Literature shows that feeding animals according to their needs is possible but poses a greater burden on the management (Holle and Rahmann 2006; Kluth 2015; Ökolandbau 2020c). The supply with protein feed gets even more challenging as the new implemented EU regulations demand a 100% organic feed ration, exceptions are just allowed for young hens till 2026 (EU-ÖKO-VO 2018). Therefore, this life phase received a rating of 2. In the current size of organic agriculture, the higher demand for resources is not a major concern, especially with its positive environmental effects, but with the targeted further expansion of organic agriculture, it may become an issue that needs to be addressed.

##### Hot spot in the life phase feeding

- ***Land use (6):*** The higher demand on resources is caused by lower yields (De Ponti et al. 2012; Ponisio et al. 2015; Seufert and Ramankutty 2017), longer fatting periods, and less efficient feed conversion (LfL n.d.; Bellof and Schmidt 2006).

#### Animal health—rating 3 (high relevance)

The health of farm animals is influenced by a variety of factors, such as housing conditions, feeding, breeding, disease prevention and treatment, and animal care and health management (FiBL 2005; BMEL 2020; foodwatch, Tierärztliche Vereinigung für Tierschutz e.V. 2017). Animal health (physical health state) represents an important aspect of animal welfare, besides the areas of well-being and natural behavior (Knierim 2016; Tierärztekammer Berlin 2017; BLE 2020). Thus, animal health has to be seen as an important part of the societal expectations toward animal husbandry (Zander et al. 2013; WBA 2015), as a high level of animal welfare cannot be achieved without animal health.

The prevention of disease is an important basic principle and includes the selection of suitable breeds, which are adapted to the husbandry conditions of organic farming and are characterized by resistance and longevity. Furthermore, attention must be paid to appropriate hygienic housing, appropriate stocking density, and high-quality feed in terms of disease prevention (EU-Öko-VO 2018).

For animal treatment, herbal and homeopathic remedies are to be used as the first choice. According to veterinary indications, chemical or synthetic medicines can also be administered but are restricted to a maximum. Also, in organic agriculture, several vaccinations are administrated to the hens. Vaccination is an imported tool for disease prevention, especially in organic agriculture where just a restrictive use of medication is allowed (Kreyenbühl 2019). The practice of non-curative interventions such as beak trimming was not performed in organic poultry farming long before the general agreement on banning this practice in Germany came into place in 2017 (Bioland 2019; Demeter 2020; Naturland 2020). These preventive approaches have positive effects for example on the feather condition which is mostly better under organic management (Sherwin et al. 2010; Riber and Hinrichsen 2016), whereas health’s impairments such as keel bone deformities (Wilkins et al. 2011; Riber and Hinrichsen 2016), parasite infection (Sharma et al. 2019; El Jeni et al. 2021), and mortality (Weeks et al. 2016; March et al. 2019) are often not better than on conventional farms. Since animal health is an essential factor for animal welfare and does not seem to reach always a better level compared to conventional farming overall, this life phase was rated as highly relevant (3), especially for organic animal husbandry, which strives for high animal welfare standards.

##### Hot spots in the life phase animal health

- ***Breeds/genetic lines used (6):*** The use of high-yielding breeds places greater demands on the management of feeding and keeping conditions in organic farming (Le Bris 2005; Pieper 2010; Breker and Thiele 2014). Outdoor access and strict regulations on feeding and the use of medicines present further challenges for keeping these breeds healthy in organic farming (FiBL 2005; Sundrum et al. 2005; Schmidt and Bellof 2008; Frölich 2017).
- ***Education and training (6):*** Health management is crucial for animal health. Therefore, education and training are of great importance in this area (Gauly and Kaufmann 2010; Brenninkmeyer and Knierim 2015; March et al. 2019; Wang et al. 2020). This is especially true in organic farming, as the use of medicines is strictly limited by the regulations.
- ***Animal welfare (9):*** “Animal health” represents an important criterion in animal welfare. Diseases and injuries are usually accompanied by suffering and represent a serious impairment to animal welfare. Despite the preventive principle in organic farming and the more animal-friendly husbandry system, studies show mostly the same and, in some areas, a slightly inferior and in others a somewhat better health state in organic poultry farming (Jansson et al. 2010; Bestman and Wagenaar 2014; March et al 2019; Wang et al. 2020). March et al. (2019) compared six studies: In 29% of the compared pairs, organic management performed better, and in 36% of the comparisons, conventional management performed better. The latter concerns primarily parasite infestation and mortality. But also advantages in terms of well-being can be seen in organic farming (March et al. 2019). By observing 107 organic laying hen farms in eight European countries, Jung et al. (2020) found a mean mortality rate of 5.7%, a mean prevalence of food pad lesion of 30.5% per flock, a mean prevalence of keel bone damage of 44.5% per flock, and 57.3% of the flocks which showed a high parasite burden. The authors of this study concluded that there are options for improvement in the health state of organic laying hens, as a large variations between flocks were observed. If we examine the animals’ health in more detail according to specific indicators, the following was found:
- Plumage condition: Seems to be better in organic systems to a certain extent (Sherwin et al. 2010; Riber and Hinrichsen 2016). Lambton et al. (2010) as well as Bestman and Wagenaar (2014) found a positive correlation between the use of the outdoor run and feather condition. In contrast, Knierim et al. (2008) could not detect a significant difference in feather scores in conventional and organic hens.
- Keel bone fractures and deviations are a widespread welfare problem in laying hens, both in conventional and in organic farming. Studies mostly show no significant difference in the prevalence of this welfare impairment between different husbandry systems (Staack et al. 2009; Sherwin et al. 2010; Wilkins et al. 2011; Riber and Hinrichsen 2016). Jung et al. (2019) found a wide range of keel bone fractures of 3–88% per flock in 107 organic laying hen flocks.
- Foot injuries: Riber and Hinrichsen (2016) found no difference in the risk of foot injuries between organic and barn husbandry systems. The prevalence found on organic farms ranged from 5 to 30% of hens per flock with foot pad damage (Bestman and Wagenaar 2014; Hinrichsen et al. 2016; Brenninkmeyer and Knierim 2015; Jung et al. 2020).
- Parasite infection: Wide spread in organic farming (Van De Weerd et al. 2009; Stokholm et al. 2010; Sharma et al. 2019; El Jeni et al. 2021), but also studies that found no significance to conventional farming (Jansson et al. 2010; Sherwin et al. 2013; Wuthijaree et al. 2017)
- Mortality rates appear to be higher in organic poultry farming compared to conventional farming (Stokholm et al. 2010; Leenstra et al. 2012; Weeks et al. 2016; March et al. 2019).

Van De Weerd et al. (2009) conclude that most welfare problems are not restricted to organic farms, but that in some respect, it is more challenging to maintain a good health state under organic conditions. Sundrum et al. (2004), and Kijlstra and Eijck (2006), as well as Bender et al. (2013) generally consider the level of animal health in organic farming to be comparable to that in conventional livestock production. However, organic farming achieves this level with a significantly lower use of medications (BÖLW 2018; March et al. 2019). Since organic farming claims a particularly high level of animal welfare for itself, improvements must be made in the area of animal health in some cases. The criterion “animal welfare” was therefore rated with a high relevance of 3 in the life phase *animal health*.

##### Transport–rating 3 (high relevance)

The life phase *transport* is usually a short but often unfamiliar and quite stressful period in an animal’s life due to many external influences, such as noise, temperature, vibration, and stocking density (Albert Schweitzer Stiftung n.d.; Grashorn 2010; EFSA 2011).

In the EU, regulations for the transport of animals are provided by the Animal Transport Regulations (*Tierschutz-Transportverordnung*). The regulations refer to the required space during transport, its length, and the transportability of the animals. For national transport, the duration should not exceed eight hours. Long-distance transport allows durations of up to 24 h without a break (TierSchTrV 2015; BMEL 2019). As the EU regulation for organic animal farming does not provide any further regulations on the transport of animals (EU-Öko-VO 2018), the given conditions by the Animal Transport Regulations apply equally to conventionally and organically raised animals. In the guidelines of some organic associations, the transport time is limited to 4 h and to a maximum distance of 200 km but can be exceeded in exceptional cases (Bioland 2019; Naturland 2020; Demeter 2020). For organic farms, this raises the challenge to find a nearby slaughterhouse with an organic certification. Especially for poultry, this seems to be problematic in some regions (Schaack et al. 2018). Since there are no major differences between organic and conventional animals with regard to transportation, the general problems that occur during transport are discussed in this HSA. Overall, the conditions of animal transport are repeatedly criticized by animal welfare organizations and NGOs. Reports and surveys also show commonly occurring animal welfare-relevant abuses during animal transports (Deutscher Tierschutzbund n.d.a; Fötschl 2013; Osnabrücker Zeitung 2016; Landesregierung Brandenburg 2018; Animals’ Angels 2019), which is why the life phase *transport* was given a high rating of 3.

###### Hot spots in the life phase transport

- ***Organization (6):*** There are often deficiencies in the transportability of the animals and the planning and management of the transport. Prerequisites for adequate transportation in terms of animal welfare include, for example, the animals’ health condition at the beginning of transportation; the management, such as the planning of the route; and the suitability and maintenance of the transport vehicles are also important. In practice, there are often deficiencies with regard to these influencing factors (von Holleben and von Wenzlawowicz 2008; Deutscher Tierschutzbund 2011; Fötschl 2013, Osnabrücker Zeitung 2016; Niedersächsisches Ministerium für Ernährung, Landwirtschaft und Verbraucherschutz 2019).
- ***Education and training (6):*** Good knowledge of animal behavior is essential for animal-friendly handling during transport (Gocke 2000; Neuland 2015). Also, training and education are important to raise awareness of animal welfare issues because violations of animal welfare during loading and unloading as well as during transport are regularly detected (von Holleben and von Wenzlawowicz 2008; Deutscher Tierschutzbund 2011; Fötschl 2013; Osnabrücker Zeitung 2016; Niedersächsisches Ministerium für Ernährung, Landwirtschaft und Verbraucherschutz 2019).
- ***Product quality (6):*** Injuries and stress during transport can affect meat quality (Maak et al. 2003; Ristic 2011; Knierim et al. 2016).
- ***Animal welfare (9):*** In connection with transport, there are a series of animal welfare problems. The catching and loading of poultry is a very stress-related part of transportation (Krautwald-Junghanns 2021). Lund et al. (2013) found that 74.2% of broilers who arrived dead at the abattoir had died due to inadequate pre-slaughter handling. The transportation of sick animals and non-transportable animals (for example, those with injuries or in a poor general condition) is a violation of the Animal Transport Regulations (*Tierschutz-Transportverordnung*). Nevertheless, animals that are not fit for transport are frequently transported regardless of their constitution (von Holleben and von Wenzlawowicz 2008). For these animals, the stress of transport is often associated with pain and suffering. Herr (2016) found significant higher losses at transport in flocks from indoor systems which had an overall poorer health condition compared to free-range flocks. Animal welfare problems during transport may further occur in the form of heat and cold stress, hunger and thirst, restricted movement, pain, and exhaustion (EFSA 2020; Landesregierung Brandenburg 2018; Rydzik 2020; Krautwald-Junghanns 2021). Especially a too high stocking density can increase the stress for the animals. Overloading of animal transports is often detected and criticized during inspections and can lead to stress and death (Animals’ Angels n.d.; Landesregierung Brandenburg 2018; topagrar 2018a; b; c; agrarheute 2019; Polizeidirektion Chemnitz 2020; Aachener Zeitung 2020).
- Transport-related animal losses are a fixed statistical variable in poultry and are defined by the so-called death on arrival (DOA) rate. This rate should not exceed 0.2% (Rabitsch 2014)–0.5% (Holmes 2020) of the total number of transported animals. However, there are currently no legally binding requirements for a limit value (Holmes 2020). By reviewing four studies, Herr (2016) found DOA rates for laying hens ranging from 0.27 to 2.5%. In particular, transportation at extreme outdoor temperatures leads to higher death rates during transport in poultry (Gocke 2000; Petracci et al. 2006; Voslarova et al. 2007). Also, the length of the transport route influences the death rate (Voslarova et al. 2007; Holmes 2020; Krautwald-Junghanns 2021). Since there are no major differences in transport between organic and conventional farming, organic farming cannot distinguish itself from the general deficits and thus this life phase received a high rating of 3 here. This applies in particular because organically raised animals can have even longer transportation distances due to the need of a slaughterhouse with organic certification (Kurier 2016; bio Press 2017; Schaack et al. 2018).

##### Slaughter–rating 3 (high relevance)

The life phase *slaughter* includes the arrival of the animals at the slaughterhouse, and their stay in the waiting area, as well as the stunning and killing process.

As with transport, there are generally no special regulations for the slaughter of organic animals (EU-Öko-VO 2018). Although abattoirs that slaughter organic animals require organic certification, this only relates to the separate slaughter and processing and does not contain any specifications for the slaughter process itself (Ökolandbau 2018c, Ökolandbau 2020d). Therefore, only the EU-wide provisions of the regulation on the protection of animals at the time of killing (*Tierschutz-Schlachtverordnung*) apply to organic animals. This regulates the handling and care of animals at the slaughterhouse, as well as the use and application of stunning methods. For poultry, stunning by means of electric baths, electric forceps, and CO_2_ stunning (except for waterfowl) is permitted (TierSchlV 2012). From an animal welfare point of view, CO_2_ stunning is currently seen as the preferable method for poultry, as the animals are not exposed to any stressful handling prior to stunning (Von Wenzlawowicz 2019; Holmes 2020). However, this stunning method also has disadvantages because the CO_2_ has an irritating effect on the mucous membranes of the animals, which causes stress and anxiety (Hänsch 2009; Zvonek 2017). Regardless of the applied stunning method, the killing is done by bleeding the animal out.

In the organic sector, alternative slaughter methods, such as mobile slaughterhouses, are used and being developed further. However, the number of animals slaughtered in alternative systems is still very low (Ökolandbau 2020e, Naturverbund 2020a).

In the slaughter industry, there has been an increasing concentration of large-scale operations in recent years. The high cost pressure and strict hygiene requirements have led to mergers and acquisitions (WBA 2015).

For poultry, some small- and medium-sized organic certified slaughterhouses exist (Ökolandbau 2020d). Overall, however, there is a lack of smaller regional slaughterhouses, especially for laying hens. Many spent hens are slaughtered in large conventional poultry slaughterhouses. Out of a calculated total slaughter volume of 4800 t organic spent hens in 2016, about 2200 t were not marketed as organic (Schaack et al. 2018).

In some slaughterhouses with organic certification, working conditions appear to be better at some sites. For example, the slaughterhouse Naturverbund Thönes pays its employees a fixed wage and does not use piecework (Naturverbund 2020a; Wirtschafts Woche 2020), and GETI WILBA employs only a few people as temporary workers (Niederelbe-Zeitung 2020). However, especially in the large conventional poultry slaughterhouses in which organic spent hens are often slaughtered, the working conditions are characterized by the extensive use of work contracts, as is common in the meat industry (DGB 2020).

Overall, the reputation and standing of the German slaughter industry can be described as poor (Albersmeier and Spiller 2009). Due to the recent COVID-19 outbreak in some slaughterhouses in the summer of 2020, the poor working conditions in the meat industry have been heatedly discussed by the public. For a long time, there have been deficits in labor and animal welfare in the industry, which have not been significantly improved until today (Deutscher Tierschutzbund 2010; DGB 2020; Der Spiegel 2020). Therefore, the life phase *slaughter* was rated with a high relevance of 3.

###### Hot spots in the life phase slaughter

- ***Used equipment/training (6):*** Poor maintenance of the equipment and training of personnel lead to animal welfare problems during slaughter (Niedersächsisches Ministerium für Ernährung, Landwirtschaft und Verbraucherschutz 2019; Osnabrücker Zeitung 2020; EFSA 2020).
- ***Water demand and pollution (9):*** The water consumption occurring in the slaughter and subsequent further processing can be described as high (Westfleisch 2010; Umweltbundesamt 2013). According to current hygiene regulations, drinking water must be used in almost all washing and rinsing processes, which severely limits the reuse of water (Westfleisch 2010; Tier-LMHV 2021). The Märkische Geflügelhof-Spezialitäten GmbH, which slaughters an average of 160,000 chickens per day, has a daily water consumption of 1.5 million liters (396 million liters per year) (Landtag Brandenburg 2017). Further, contact with carcasses or animal by-products causes the water to become heavily contaminated with organic substances, such as blood, hair, fat, or feces, which makes cleaning the wastewater challenging (Abwasser Analysezentrum (n.d.); Umweltbundesamt 2013). The cleaning of facilities with detergents and disinfectants also pollutes the wastewater (Umweltbundesamt 2013). Water consumption as well as its pollution during slaughtering can thus definitely be considered an ecological hotspot (Zühlsdorf and von Meyer-Höfer 2011), which must be mitigated as much as possible.
- ***Education and training (6):*** Most animal welfare problems at slaughter are directly related to human activity. Despite the required certificates of competence, slaughterhouses often disregard animal welfare regulations, and stunning techniques are applied improperly (Fötschl 2013; EFSA 2020).
- ***Working conditions (9):*** Working conditions in the German slaughter and meat industry have long been criticized (Niedersächsische Landesregierung 2004; FAZ 2013; Weinkopf and Hüttenhoff 2017). The COVID-19 outbreaks (summer 2020) in some large German slaughter companies brought the existing abuses in the industry into the light of public discussion once again (Braunschweiger Zeitung 2020; FAZ 2020; tagesschau 2020). Since the 1990s, the German meat industry has been characterized by the extensive use of service contracts (Werkverträge) (DGB 2020). Brinkmann and Nachtwey (2014) reported on the extensive use of these service contracts in slaughterhouses, which amount to about 90% of the employment relations. The outsourcing of work steps means that the slaughter company itself bears no responsibility for the workers and that there is no control over the payment of fair wages and compliance with working hours, and health and safety guidelines cannot be guaranteed (FrankfurterRundschau 2013; FAZ 2013; Brinkmann and Nachtwey 2014; Weinkopf and Hüttenhoff 2017; DGB 2020). Also, the German Institute for Human Rights denounces the severe labor exploitation of migrant workers in the meat processing industry (Deutsches Institut für Menschenrechte 2018). In response to pressure from widespread public reporting, the “Occupational Safety and Health Program for the Meat Industry” was implemented to improve working conditions in the meat industry (Bundesregierung 2020). These problems mostly exist in large “conventional” slaughterhouses. Nevertheless, organic laying hens often end up in these abattoirs, especially those of larger units (Schaack et al. 2018). Thus, there are serious deficits in the areas of general working conditions, social security, income, and occupational health. As a result, these criteria are considered hotspots (rating 3) in the life phase *slaughter*.
- ***Product quality (6):*** Stress and injuries during the slaughter process reduce meat quality (Fötschl 2013; Naturverbund 2020b).
- ***Animal welfare (9):*** Animal welfare problems occur in all areas in connection with slaughtering. Serious violations of animal welfare exist from the delivery of the animals, and their handling while being brought to the stunning facilities, as well as during stunning and bleeding (Deutscher Tierschutzbund 2010; Fötschl 2013; Albert Schweitzer Stiftung 2019a; EFSA 2020). Small- and medium-sized abattoirs are just as affected (Albert Schweitzer Stiftung 2019b) as large ones (Reymann 2016), and animal welfare violations are also found in slaughterhouses with organic certification (Albert Schweitzer Stiftung 2019a). Upon arrival at the slaughterhouse, extended waiting times can cause animal welfare problems in the form of heat and cold stress, hunger, and thirst, restricted movement, and pain, as well as exhaustion (EFSA 2020). Greshake (2018) reports regular delays in the transporting and unloading of the animals. Often, the capacity of the available waiting rooms at the slaughterhouse is very limited, which means that the animals have to wait on the transporters. In particular, poultry delivered in transport containers cannot be adequately cared for if there are delays at the slaughterhouse or during transport (Consortium of the Animal Transport Guides Project 2017). In some cases, long waiting times accrue for injured animals, which often leads to the animals’ death prior to slaughter (Fötschl 2013). Also, the animals are frequently not even separated or killed on the spot but are brought to the stunning facility while they are in pain, to ensure that there are no delays in the slaughter process (Fötschl 2013; Deutscher Tierschutzbund 2011; Niedersächsisches Ministerium für Ernährung, Landwirtschaft und Verbraucherschutz 2019). Some of the most serious animal welfare problems at the slaughterhouse occur in connection with stunning and bleeding. While water bath stunning can cause electric shocks and pain when the animals are hung in the stirrups and immersed in the water basin (von Holleben and von Wenzlawowicz 2006; von Wenzlawowicz 2019; EFSA 2019), CO_2_ stunning causes irritation of the mucous membranes as well as feelings of suffocation in the animals (Hänsch 2009; Machtolf et al. 2013; EFSA 2020). For either method, no reliable numbers on stun failure rate are available. In other animal species, insufficient stunning is often seen. For example, during official inspections in 31 slaughterhouses in the Darmstadt administrative district (2014–2017), nearly 50% of the cattle, 45% of the sheep, and 39% of the pigs showed signs of regaining consciousness after stunning. In 61 animals, movements could still be detected during further processing. In addition, bleeding was too slow in 17% of the animals (Albert Schweitzer Stiftung 2019b). These not infrequently occurring errors in stunning and bleeding (Deutscher Tierschutzbund n.d.b; Deutscher Bundestag 2012; Fötschl 2013; Osnabrücker Zeitung 2020) are associated with extreme suffering for the animals, especially if further processing takes place before death occurs due to high slaughter speeds (Fötschl 2013; Troeger 2014; Albert Schweitzer Stiftung 2019b). In practice, the success of bleeding is usually only verified on a random basis. Large slaughterhouses such as Tönnies have already introduced an automatic weighing control before and after sticking in order to be able to monitor the bleeding success (Hünerfeld 2014). In view of a large number of frequent animal welfare violations at slaughterhouses, compliance with the German Regulations for Animal Protection and Slaughter and the German Animal Welfare Act must be consistently monitored, and non-compliance must be punished. Currently, it cannot be assumed that all animals in German slaughterhouses are killed without pain and suffering. Therefore, animal welfare was assessed as a hotspot (rating: 3) in the life phase *slaughter* in this study.

Tabe 3 shows the identified hot spots in organic laying hen husbandry. As an overview to the detailed ratings, a table displays all the total ratings with numbers in the Appendix (Table 4).

**Table 3:** All identified hotspots for organic laying hen husbandry according to the associated life phases and criteria

Life phases	Breeding	Keeping	Feeding	Animal health	Transport	Slaughter
Ecological criteria						
Breeds/genetic lines	Breeding process: monopoly, dependency	–	–	Breeds/genetic lines used	Organization	Used equipment/training
Energy	–	–	–	–	–	–
Greenhouse gas emissions	–	–	–	–	–	–
Water consumption	–	–	–	–	–	High use of water
Land use	Dual purpose breeds: higher requirement of resources	–	Higher requirement of resources	–	–	–
Biodiversity	Loss of genetic diversity	–	–	–	–	–
Water, soil and air emissions	–	High N emissions*	–	–	–	Water pollution
“By-products”	male layers	–	–	–	–	–
Life phases	Breeding	Keeping	Feeding	Animal health	Transport	Slaughter
Social criteria						
Working conditions	–	–	–	–	–	Labor exploitation in slaughter companies
Social stability	–	–	–	–	–	
Workers ‘ health	–	–	–	–	–	
Human rights	–	–	–	–	–	
Income	–	–	–	–	–	
Education and training	–	–	–	Education and training are essential for animal health and animal welfare		
Consumers ‘ health	–	–	–	–	–	–
Product quality	–	–	–	–	Stress and injuries decrease product quality	
Animal welfare						
Animal protection	Breeding-related health disorders, culling of male chicks from layers	–	–	No better health state than in conventional poultry farming	Violations against animal transport regulations	Violations against regulation on the protection of animals at the time of killing

Source: own description

*Problematic areas which do not represent a hotspot, for example, because the life phases received a low rating. In any case, they must be considered an environmental problem

After the hotspots were identified based on a literature review, the results were discussed with different experts.

### Results of expert interviews

The identified hotspots were discussed with nine different experts. Table 5 in the Appendix gives an overview of their characteristics and type of expertise. The experts interview ID indicates which statement is assigned to which experts. The main questions are presented below.

#### Would you change any of the assessments and have we missed an important point?

Overall, the hotspots found through the literature research coincide well with the experts’ opinions. Experts also saw especially the upstream and downstream areas as hotspots and as possible risk points to not meet consumers’ expectations (I1, I2, I4, I5, I6, I7, I8, I9). There were lighter discrepancies in the ratings of the life phases *keeping* and *animal health*. Four of the 9 interviewed experts said they would rate the life phase *keeping* with a higher value than 1 (I3, I4, I5, I8). Reasons given for this were that the minimum standards of the EU regulations for organic animal farming are still too low for a good animal welfare level and also do not include regulations on herd and group sizes (I4, I5, I7, I8).

Three experts (I1, I8, I9) stated that they would rate the area of animal health somewhat lower. They noted that it should be differentiated by which health concept is referred to (I9). A light parasite load cannot be considered a disease and belongs, to some extent, to a natural husbandry system (I8).

There were no major discrepancies among the answers provided by the various expert groups (science, opinion leaders, and practice).

#### Which hotspots would you classify as the ones most at risk of losing consumers’ trust?

Experts see in particular the upstream and downstream areas (breeding and transport/slaughter) as critical areas for loss of trust due to grievances and lack of specific organic standards (I1, I2, I4, I5, I6, I7, I8, I9). Regarding husbandry, especially stock sizes () and animal health (I2, I3) are seen as a risk for the loss of consumer trust in organic livestock production. In breeding, particularly the killing of day-old male chicks of layers can disappoint consumers (I9, I4, I6). However, it was also highlighted that most consumers have very little knowledge on this topic (I1, I4, I6). Almost all experts rated the areas of transport and slaughter as a possible trust hotspot (I1, I2, I4, I5, I6, I7, I8, I9). The general grievances in this area pose a particular problem for organic livestock production, as it cannot distinguish itself from the conventional system here, and consumers could expect better conditions (I1, I4, I6). Further, it was mentioned that it is difficult and costly for the organic sector to enforce its own standards in this area, as it is a structural problem (I1, I4). Here, too, consumers are often not aware of the grievances (I4, I8, I9). Some experts also considered reports on poor animal health in organic farming to be a possible risk to consumer trust (I2, I3). With regard to husbandry, management deficiencies (I3) and the large poultry stocks (I5, I7) on some organic farms were seen as risk points for a loss of consumer trust.

## Discussion

### Classification of the life phases as potential risk to consumer trust

The results show that especially the life phases *breeding* and *slaughter* show a number of problematic areas. There are also some weaknesses in the life phases *animal health* and *transport*. In contrast, the life phases *keeping* and *feeding* show no or only single hotspots due to their low rating with regard to sustainability weaknesses. Nevertheless, there are also ecological deficits and challenges in animal welfare in these life phases.

In the following, an initial classification of the life phases with their sustainability weaknesses is made with regard to a potential risk of losing consumer trust. However, a distinction must be made here between the hotspots in the area of sustainability and a potential trust risk. Some hotspots, such as high-water consumption and water pollution during slaughter, can be considered a low trust risk compared to animal welfare hotspots, due to a lower emotionality of the topic. However, the partly large farm structures of organically raised poultry of up to 30,000 animals can entail a high potential for the loss of consumer trust, even when the life phase *keeping* itself received a low rating.

#### Classification of the life phase breeding as a potential risk to consumer trust

Generally, breeding is a problematic area in organic animal husbandry that needs to be discussed. The use of one-sided specialized breeds is difficult to reconcile with the basic idea of a holistic approach. From a social point of view, highly specialized poultry breeding also poses problems in terms of power distribution and access to animal genetic resources, and genetic diversity. On the other hand, the existing problems in this field must always be seen under the given framework conditions since there is often a lack of economically profitable alternatives to the specialized breeds in organic farming. In addition, it is not easy to implement one’s own demands in practice, since, for example, dual-purpose chickens are currently only of small value on the market (Ökolandbau 2018a). Recently, the organic sector has been making efforts to implement its own breeding structures. Breeding companies are also increasingly bringing breeds to the market that can be reconciled with the ideas of organic animal husbandry. Breeding itself seems to be a rather small present subject to many consumers. The interviewed experts as well as Hörning (2013) suspect a rather low level of consumer knowledge about animal breeding. That is why, currently, mainly individual aspects of animal breeding can be considered risk points for the loss of trust in organic animal husbandry. In particular, topics that are publicly discussed, such as the killing of male chicks of laying hens (which is prohibited since 2022) represent such a trust risk.

#### Classification of the life phase keeping as a potential risk to consumer trust

Even though the life phase *keeping* was rated with a low overall relevance with regard to sustainability problems (high keeping standards, limited stocking density per ha) in this HSA, it does hold potential for a loss of trust. The more animal-friendly keeping conditions in organic farming can lead to high expectations among consumers. If deficiencies in animal husbandry occur in practice, for example, due to poor management, this can lead to consumer disappointment. Further, the requirements for a housing system that is particularly oriented to the needs of the animals, with more space and outdoor access, carry some challenges and dangers in keeping the animals healthy, such as predators and an increased risk of disease (Brenninkmeyer et al. 2013; March et al. 2019). However, outdoor access and thus the ability to perform species-specific behaviors has a positive effect on the animals’ well-being. In this respect, it would be interesting to know how consumers perceive and evaluate this conflict of interest, as it is known that outdoor access seems to be the ideal standard for animal keeping (Kühl et al. 2019). However, it should be noted that, due to the lack of consumers’ knowledge, acceptance cannot represent a measure of the animal welfare of husbandry systems. Rather, welfare should always be based on the animals’ needs (Schmidt 2020).

Moreover, the partly large farm sizes, especially in the poultry sector of up to 30,000 animals per farm (Statistisches Bundesamt 2018), often do not match the expectations of consumers. Especially in the organic sector, consumers expect small farms and stock sizes (Vaarst and Hovi 2004; Chang and Zepeda 2005; Meas et al. 2014).

With the alignment toward conventional structures, the “David-vs-Goliath” picture gets damaged and holds the risk of losing favorable attributes some consumers may ascribe to organic farming. In general, organic agriculture aims to represent an alternative to conventional agriculture, but it cannot serve this aim when it takes over the same structures (De Wit and Verhoog 2007).

This problem is particularly relevant on farms that farm solely according to EU regulations, as the associations impose far greater restrictions on the number of housing units per building (Bioland 2019; Naturland 2020; Demeter 2020). It should further be noted that the flock size and stocking density are more relevant aspects from an animal welfare perspective than the overall farm size but this is hardly perceived by consumers.

##### Classification of the life phase feeding as a potential risk to consumer trust

The increased land requirements for feed production in organic farming are a sustainability hotspot. The higher demand for resources is caused by lower yields, longer fatting periods, and less efficient feed conversion. However, the different approach of organic farming must also be considered, which represents a holistic system and is not specifically geared toward the efficient production of animal products, but rather sees arable farming and livestock farming as a cohesive unit. Strict organic regulations imply lower input levels in organic feed production and thus bring a number of ecological benefits, such as higher biodiversity and efficient reuse of nitrogen (Chmelikova and Hülsbergen 2019; Stein-Bachinger et al. 2019). Therefore, a wide expansion of organic agriculture with less efficient animal production can only be accompanied by reduced consumption of animal products (Muller et al. 2017). Most interviewed experts did not see the higher demand on resources in organic livestock farming as a risk point to disappoint consumers at the moment. The approach of a holistic system is comprehensible and easy to communicate to consumers. One point of consumer disappointment in organic feeding may be feed imports over long distances (BIOAktuell.ch 2015). With regard to consumer expectations, on the other hand, the clear ban on genetic engineering in organic feeding is positive (Kubitzki et al. 2009; PwC 2017). Aspects of needs-based feeding and its assessment seem to be a rather too specific topic for consumers.

##### Classification of the life phase animals’ health as a potential risk to consumer trust

In the area of animal health, organic farming imposes strict regulations regarding the type and frequency of medications used. Therefore, good health management is particularly necessary for organic animal husbandry. In many areas, the same level of health as in conventional livestock production can be achieved with significantly lower use of medications, despite the additional health risks posed by free-range farming (March et al. 2019). Animal health as an important component of animal welfare is of great importance for organic farming. Organic livestock farming aims to represent a high animal welfare level. In almost all areas of animal husbandry, management plays a crucial role in animal health (Sundrum et al. 2004; Gauly and Kaufmann 2010; Brenninkmeyer and Knierim 2015; March et al. 2019; Wang et al. 2020). The management might even have a higher impact on animal health than the husbandry system (Wang et al. 2020). This underlines the importance of education and training in this field and shows the influence of engagement of the animal keeper.

The majority of health impairments are multifactorial, so management errors in husbandry, feeding, and health care are often reflected by poor animal health, which is clearly contrary to consumers’ expectations. This is especially true for visible health problems, such as feather pecking and cannibalism, but also non-obvious diseases can represent a potential loss of consumer trust through reporting (Rahmann and Oppermann 2005; Werner et al. 2008; Weiler et al. 2009; PwC 2017; foodwatch 2019). A poor state of animal health as a risk point to disappoint consumers was also mentioned by some of the interviewed experts.

Studies show that naturalness plays an important role in the perception of organic animal husbandry (Christoph-Schulz et al. 2018; Cardoso et al. 2019). With regard to the more natural husbandry conditions of organic farming and its associated health risks (e.g., predators or parasite contamination) (March et al. 2019), the question emerges to what extent consumers might accept slightly poorer animal health in this area since a certain degree of disease and danger often goes along with the natural lifestyle (Lund 2006). This should be the subject of further research. Nevertheless, in the interest of animal welfare, the overall aim should always be to reduce all kinds of impairments and diseases to a minimum.

##### Classification of the life phases transport and slaughter as a potential risk to consumer trust

In the life phases of *transport* and *slaughter*, there are no specific regulations for organic animals provided by the organic EU regulations. Since the life phases of *transport* and *slaughter* often have serious deficits, especially in labor and animal welfare, this can pose a grave trust problem for organic animal husbandry. Organic certification of slaughterhouses may also contribute to this. This certification can easily be associated with higher standards in the slaughter of animals, but only refers to the separate processing and labeling process. The slaughter industry is characterized by a high degree of concentration (WBA 2015; Greshake 2018; Ökolandbau 2020d). In some regions, this leads to even longer transportation distances (Schaack et al. 2018), which may not be in line with consumers’ expectations. Even stress-free forms of slaughter originating from organic farming, such as bullet shooting on pasture, are currently only approved under strict conditions in Germany (agrarheute 2020). Despite these difficult conditions, organic agriculture should pay greater attention to the areas of transport and slaughter and formulate its own criteria and standards here as well. In view of the existing problems, it is difficult to communicate to consumers that organic standards contain precise specifications for all areas, such as breeding, keeping, animal health, and feeding, but then disregard the final steps of transport and slaughter, which are often characterized by animal welfare problems. The transport and slaughter of organically raised animals can therefore be seen as one of the biggest areas for the loss of trust in organic livestock production. This is in line with the assessment of the interviewed experts, who attributed a huge potential for consumers to be disappointed in these life phases.

### General classification of the HSA

If we look at organic livestock farming in general, there are always some approaches to solve the problems described here, either at the farm level or through larger initiatives, such as the Ökologische Tierzucht GmbH in breeding or in slaughtering, with the development and promotion of slaughter mobiles and pasture shooting. However, these approaches are not yet widely used in organic livestock production. As a result, it is difficult to take a general view on organic animal husbandry, as the EU regulations on organic production represent just the lowest common denominator, and there is a high degree of variability between farms (Animal Health Online 2016; WBAE 2020). Both exemplary and less committed farms can be found in all areas. This HSA focuses heavily on the critical issues in order to identify as many of the existing problems as possible. It must be considered that the hotspots do not exist on all organic poultry farms to the same extent, and on some, they do not exist at all. Many farms try to circumvent and improve the existing structural problems, such as those in breeding, transport, and slaughtering, with their own farm-specific solutions and initiatives.

If one wants to assess organic farming, the history of organic farming must also be taken into account. It developed as an alternative to conventional farming (BÖLW 2012), which means that there is far less research in all areas. Furthermore, many problematic areas need to be considered in a differentiated way. For example, in slaughtering, large companies in particular stand out negatively for tolerating exploitative working conditions in their companies, and the high slaughter numbers can have a negative impact on the stress level and the handling of animals as well. However, precisely these large companies are able to finance technology and personal to check the success of bleeding. Due to economic constraints, however, smaller and medium-sized slaughterhouses cannot always guarantee this.

The weak points presented here must always be seen under the given framework conditions, such as existing economic constraints or legal regulations. For example, the currently common CO_2_ stunning of pigs in larger slaughterhouses is used as a cheap and effective form of stunning despite serious animal welfare concerns. As with the long-tolerated practice of chick killing, such existing abuses in animal husbandry may one day be deemed unacceptable by society. For organic animal husbandry, which plays a pioneering role with regard to animal welfare, it is therefore of particular importance to look for possible alternatives at an early stage in order to circumvent the existing problems. In areas where organic farming cannot meet its own requirements in practice, it runs the risk of losing credibility and trust among consumers. Organic farming should continue to pursue its pioneering role in environmental protection and animal welfare by recognizing existing problem areas and by constantly improving them, as well as by involving consumers.

### Recommendations for action

In the following, some recommendations for action are discussed for the identified hotspots. Due to economic and structural dependencies, it is difficult for organic animal husbandry to put its own demands into practice, especially in the upstream and downstream areas (breeding/transport and slaughter). But there are some promising approaches to circumvent existing problems, like the Ökologische Tierzucht GmbH, RegioHuhn, ÖkoGen or alternative slaughter methods. Further, organic agriculture is leading the way in the area of management control with the development and application of animal-related indicators to measure animal welfare (AG Tierwohl 2014). These initiatives should be pursued further and expanded in the interest of animal welfare and to improve the credibility of organic agriculture. With regard to some weak points in the areas of husbandry, feeding, and animal health, an adjustment of EU regulations for organic animal farming could reduce some existing challenges. On the one hand, a much stricter limitation of animal numbers in the poultry sector would be conceivable, as there are sometimes very large stocks that are difficult to reconcile with the idea of organic poultry farming. On the other hand, the relaxation of the requirements with regard to synthetically produced methionine in feeding and the abolition of the maximum medical treatment frequency of an animal could be conceivable approaches to facilitate animal welfare-oriented husbandry and feeding. Furthermore, the currently almost completely missing regulations for transport and slaughter should be adjusted in the organic regulation. Due to the currently widespread abuses in terms of labor and animal welfare during transport and slaughter, the organic sector overall should pay more attention to these areas. The regulations mentioned in the guidelines of the organic associations of a maximum transport distance of 200 km and a duration of 4 h could also be included in the EU’s regulations for organically raised livestock. Since it is not possible for every organic farm to find a suitable slaughterhouse within this radius, exceptions should be permissible. Furthermore, linking the organic certification of a slaughterhouse to animal welfare standards during slaughter would be recommended as a consistent step. The regulations on transport and slaughter of the German Animal Welfare Association could serve as an orientation for this (Deutscher Tierschutzbund 2021). Currently, organic certification, which only refers to separate processing and labeling, can easily lead to misunderstandings among consumers.

The following recommendations for action are addressed to policymakers.

Compliance with the existing regulations must be monitored much more strictly to minimize existing abuses in the areas of transport and slaughter in the future. A nationwide uniform control system with effective sanctions is needed. Responsible veterinarians and police officers should be made more aware of animal welfare problems through special training and need to be educated in this area. In addition, some regulations of the Animal Welfare Transport Ordinance are too weak, and the wording is often too vague, which is why their interpretation can vary greatly. Usually, the regulations are interpreted to favor economic reasons, not animal welfare. To ensure a reliable protection of animals during transport, existing regulations with regard to space allowance, transport duration, and maximum temperatures need to be tightened (Deutscher Tierschutzbund n.d.a; Animals’ Angels n.d.; Höfken 2018; Aachener Zeitung 2020).

Alternative slaughtering methods, such as pasture shooting or on farm slaughtering, as particularly animal-friendly slaughtering systems should also be supported more strongly by politics. In this context, the progressive centralization in the slaughter industry should be counteracted. A slaughterhouse that is as close as possible to the farm is a fundamental prerequisite for carrying out farm or pasture slaughter or for simply shortening transport distances. The COVID-19 crisis also highlights the weaknesses of a reliance on a few large companies. Reductions in slaughtering due to COVID-19-related staff absences in some large slaughterhouses resulted in a backlog of pigs throughout the system (BWagrar 2020; WirtschaftsWoche 2021). Overall, animal welfare should be given a much higher priority during slaughter. For example, research into alternatives to CO_2_ stunning must be promoted and advanced much more strongly; in the case of pigs in particular, this stunning method does not comply with the requirements of the Animal Welfare Act, as the animals are not stunned without pain and suffering. Also, water bath stunning in poultry is not considered in compliance with animal welfare requirements (EFSA 2019, 2020). Thus, the currently most common stunning methods are not in line with the Animal Welfare Act in Germany.

The ban on service contracts in the meat industry can be seen as a first step to improve working conditions in the industry. However, policy makers should ensure that working conditions improve fundamentally and sustainably in the long term. With regard to the expansion of organic farming (target: 20% organic farming by 2030), research for organic farming should also be promoted more strongly. Compared to conventional agriculture, there is a high research deficit here with regard to suitable varieties, cultivation methods, husbandry practices, and technology.

## Limitations

Limitations of this study are, in particular, the authors’ subjective perspective regarding the evaluation of the life phases and criteria on the basis of a literature review. Although this is reduced by a systematic approach and by supplementing the results with expert interviews, it can never be completely eliminated. Due to the heterogeneity of the object of investigation, only a very general overview of existing problem areas in organic poultry farming can be provided. Finally, it must be considered that not all hotspots exist to the same degree on every organic farm, and for some, they do not exist at all.

## Data Availability

Not applicable.

## Appendix

**Table 4:** Total rating of all criteria for organically kept laying hens in each life phase

Life phases Ecological criteria	Breeding (3)	Keeping (1)	Feeding (2)	Animal health (3)	Transport (3)	Slaughter (3)
Breeds/genetic lines	6	0*	1	6	6	9
Energy	n.L.*	0	1	0	0	n.L
Greenhouse gas emissions	6	1	1	0	3	n.L
Water consumption	6	1	0	0	3	9
Land use	6	0	6	0	0	0
Biodiversity	9	0	1	0	0	0
Water, soil and air emissions	0	3	1	1	0	9
“By-products”	6	0	0	0	0	3
Life phases Social criteria	Breeding	Keeping	Feeding	Animal health	Transport	Slaughter
Working conditions	0	1	0	0	3	9
Social stability	0	0	0	0	0	9
Workers ‘ health	0	0	0	3	3	9
Human rights	0	1	1	3	0	9
Income	0	0	1	0	0	9
Education and training	0	0	0	6	6	6
Consumers ‘ health	3	1	1	3	0	0
Product quality	3	1	1	3	6	6
Animal welfare	Breeding	Keeping	Feeding	Animal health	Transport	Slaughter
Animal protection	9	2	4	9	9	9

*A rating of 0 means that no application of this criterion in the life phase was given. The abbreviation “n. L.” stands for “no literature” meaning that no literature for a sufficient evaluation was found.

**Table 5:** Overview of the characteristics and type of expertise of the nine interviewed experts

Interview ID	Gender	Age (shown in 5 year steps)	Type of expertise	Position
Scientists				
I1	Male	> 55	Researcher in the fields of market analysis of the organic market and marketing, management in the organic sector and sustainable food systems	Professor
I2	Female	> 60	Researcher in the fields of animal husbandry, animal health and welfare with focus on organic animal husbandry	Professor
I3	Male	> 70	Veterinarian and researcher in the field of animal health	Professor emeritus
Practitioners				
I4	Male	> 60	Farm manager of an organic laying hen farm, with new concepts such as dual-purpose breeds	Farmer
I5	Male	> 60	PhD in agricultural sciences, farm manager and consultant in the field of organic animal husbandry	Farmer, consultant
I6	Male	> 75	PhD in feeding organic laying hens, consultant for organic poultry farming	Consultant
Opinion leaders				
I7	Female	< 55	Cook and activist for animal friendly agriculture, member of the European Parliament	Politician, cook
I8	Male	> 70	Farmer and board of an animal welfare association/ trademark	Farmer, board member of an animal welfare trademark
I9	Female	> 50	Author, moderator, journalist in the fields of agriculture, sustainability, nutrition, biodiversity and animal husbandry	Author, moderator, journalist
